# Supplementary material for: Unveiling the Bioactive Potential of the Invasive Jellyfish Phyllorhiza punctata Through Integrative Transcriptomic and Proteomic Analyses
Source: Biomolecules. 2025 Aug 4;15(8):1121. doi: 10.3390/biom15081121 (PMC12383608; doi:10.3390/biom15081121)
Supplement: Supplementary file 1 [file biomolecules-15-01121-s001.zip › Suplementary Material/FileS3/PEPFOLD4/XP_065061388_14675/clusters.html]

 PEP-FOLD clusters


Download archive of all models  here   

|  |  |  |
| --- | --- | --- |
| # | file | sOPEP |
| # cluster 1 - Size: 80 |  |  |
|  | XP\_065061388\_14675-model1 | -24.7109 |
|  | XP\_065061388\_14675-model1.1 | -24.7109 |
|  | XP\_065061388\_14675-model1.2 | -24.7109 |
|  | XP\_065061388\_14675-model1.3 | -24.7084 |
|  | XP\_065061388\_14675-model1.4 | -24.7084 |
|  | XP\_065061388\_14675-model1.5 | -24.7084 |
|  | XP\_065061388\_14675-model1.6 | -24.7084 |
|  | XP\_065061388\_14675-model1.7 | -24.7084 |
|  | XP\_065061388\_14675-model1.8 | -24.7084 |
|  | XP\_065061388\_14675-model1.9 | -24.7084 |
|  | XP\_065061388\_14675-model1.10 | -24.7084 |
|  | XP\_065061388\_14675-model1.11 | -24.7084 |
|  | XP\_065061388\_14675-model1.12 | -24.7084 |
|  | XP\_065061388\_14675-model1.13 | -24.7084 |
|  | XP\_065061388\_14675-model1.14 | -24.7084 |
|  | XP\_065061388\_14675-model1.15 | -24.7084 |
|  | XP\_065061388\_14675-model1.16 | -24.7084 |
|  | XP\_065061388\_14675-model1.17 | -24.7084 |
|  | XP\_065061388\_14675-model1.18 | -24.7084 |
|  | XP\_065061388\_14675-model1.19 | -24.6934 |
|  | XP\_065061388\_14675-model1.20 | -24.6905 |
|  | XP\_065061388\_14675-model1.21 | -24.6905 |
|  | XP\_065061388\_14675-model1.22 | -24.6905 |
|  | XP\_065061388\_14675-model1.23 | -24.6905 |
|  | XP\_065061388\_14675-model1.24 | -24.6905 |
|  | XP\_065061388\_14675-model1.25 | -24.6905 |
|  | XP\_065061388\_14675-model1.26 | -24.6905 |
|  | XP\_065061388\_14675-model1.27 | -24.6905 |
|  | XP\_065061388\_14675-model1.28 | -24.6905 |
|  | XP\_065061388\_14675-model1.29 | -24.6765 |
|  | XP\_065061388\_14675-model1.30 | -24.6765 |
|  | XP\_065061388\_14675-model1.31 | -24.6714 |
|  | XP\_065061388\_14675-model1.32 | -24.6593 |
|  | XP\_065061388\_14675-model1.33 | -24.6427 |
|  | XP\_065061388\_14675-model1.34 | -24.6427 |
|  | XP\_065061388\_14675-model1.35 | -24.6427 |
|  | XP\_065061388\_14675-model1.36 | -24.6427 |
|  | XP\_065061388\_14675-model1.37 | -24.6427 |
|  | XP\_065061388\_14675-model1.38 | -24.625 |
|  | XP\_065061388\_14675-model1.39 | -24.625 |
|  | XP\_065061388\_14675-model1.40 | -24.625 |
|  | XP\_065061388\_14675-model1.41 | -24.6195 |
|  | XP\_065061388\_14675-model1.42 | -24.5869 |
|  | XP\_065061388\_14675-model1.43 | -24.5869 |
|  | XP\_065061388\_14675-model1.44 | -24.5869 |
|  | XP\_065061388\_14675-model1.45 | -24.5861 |
|  | XP\_065061388\_14675-model1.46 | -24.5861 |
|  | XP\_065061388\_14675-model1.47 | -24.5794 |
|  | XP\_065061388\_14675-model1.48 | -24.5731 |
|  | XP\_065061388\_14675-model1.49 | -24.5731 |
|  | XP\_065061388\_14675-model1.50 | -24.5731 |
|  | XP\_065061388\_14675-model1.51 | -24.5712 |
|  | XP\_065061388\_14675-model1.52 | -24.5712 |
|  | XP\_065061388\_14675-model1.53 | -24.5712 |
|  | XP\_065061388\_14675-model1.54 | -24.5712 |
|  | XP\_065061388\_14675-model1.55 | -24.5712 |
|  | XP\_065061388\_14675-model1.56 | -24.5712 |
|  | XP\_065061388\_14675-model1.57 | -24.5712 |
|  | XP\_065061388\_14675-model1.58 | -24.5712 |
|  | XP\_065061388\_14675-model1.59 | -24.5712 |
|  | XP\_065061388\_14675-model1.60 | -24.5712 |
|  | XP\_065061388\_14675-model1.61 | -24.5712 |
|  | XP\_065061388\_14675-model1.62 | -24.5712 |
|  | XP\_065061388\_14675-model1.63 | -24.569 |
|  | XP\_065061388\_14675-model1.64 | -24.5432 |
|  | XP\_065061388\_14675-model1.65 | -24.5432 |
|  | XP\_065061388\_14675-model1.66 | -24.5427 |
|  | XP\_065061388\_14675-model1.67 | -24.5408 |
|  | XP\_065061388\_14675-model1.68 | -24.539 |
|  | XP\_065061388\_14675-model1.69 | -24.5273 |
|  | XP\_065061388\_14675-model1.70 | -24.5193 |
|  | XP\_065061388\_14675-model1.71 | -24.5193 |
|  | XP\_065061388\_14675-model1.72 | -24.5052 |
|  | XP\_065061388\_14675-model1.73 | -24.4658 |
|  | XP\_065061388\_14675-model1.74 | -24.3824 |
|  | XP\_065061388\_14675-model1.75 | -24.3582 |
|  | XP\_065061388\_14675-model1.76 | -24.3387 |
|  | XP\_065061388\_14675-model1.77 | -24.3105 |
|  | XP\_065061388\_14675-model1.78 | -24.1955 |
|  | XP\_065061388\_14675-model1.79 | -24.1947 |
| # cluster 2 - Size: 10 |  |  |
|  | XP\_065061388\_14675-model2 | -20.848 |
|  | XP\_065061388\_14675-model2.1 | -20.8465 |
|  | XP\_065061388\_14675-model2.2 | -20.8186 |
|  | XP\_065061388\_14675-model2.3 | -20.8153 |
|  | XP\_065061388\_14675-model2.4 | -20.7555 |
|  | XP\_065061388\_14675-model2.5 | -20.7219 |
|  | XP\_065061388\_14675-model2.6 | -20.6889 |
|  | XP\_065061388\_14675-model2.7 | -20.6485 |
|  | XP\_065061388\_14675-model2.8 | -20.5753 |
|  | XP\_065061388\_14675-model2.9 | -20.4198 |
| # cluster 3 - Size: 2 |  |  |
|  | XP\_065061388\_14675-model3 | -14.9689 |
|  | XP\_065061388\_14675-model3.1 | -14.6981 |
| # cluster 4 - Size: 6 |  |  |
|  | XP\_065061388\_14675-model4 | -14.6904 |
|  | XP\_065061388\_14675-model4.1 | -14.6169 |
|  | XP\_065061388\_14675-model4.2 | -14.5829 |
|  | XP\_065061388\_14675-model4.3 | -14.5739 |
|  | XP\_065061388\_14675-model4.4 | -14.5456 |
|  | XP\_065061388\_14675-model4.5 | -14.4376 |
| # cluster 5 - Size: 2 |  |  |
|  | XP\_065061388\_14675-model5 | -14.58 |
|  | XP\_065061388\_14675-model5.1 | -14.5728 |
|  |  |  |
